# A sonochemical approach to the direct surface functionalization of superparamagnetic iron oxide nanoparticles with (3-aminopropyl)triethoxysilane

**DOI:** 10.3762/bjnano.5.160

**Published:** 2014-09-08

**Authors:** Bashiru Kayode Sodipo, Azlan Abdul Aziz

**Affiliations:** 1 Nano-Biotechnology Research and Innovation (NanoBRI), Institute for Research in Molecular Medicine (INFORMM), Universiti Sains Malaysia, 11800, Pulau Pinang, Malaysia and Nano-Optoelectronic Research and Technology Lab (NOR Lab), School of Physics, Universiti Sains Malaysia, 11800 Pulau Pinang, Malaysia

**Keywords:** (3-aminopropyl)triethoxysilane (APTES), functionalization, nanoparticles, silanization, sonochemical, superparamagnetic iron oxide nanoparticles (SPION)

## Abstract

We report a sonochemical method of functionalizing superparamagnetic iron oxide nanoparticles (SPION) with (3-aminopropyl)triethoxysilane (APTES). Mechanical stirring, localized hot spots and other unique conditions generated by an acoustic cavitation (sonochemical) process were found to induce a rapid silanization reaction between SPION and APTES. FTIR, XPS and XRD measurements were used to demonstrate the grafting of APTES on SPION. Compared to what was reported in literature, the results showed that the silanization reaction time was greatly minimized. More importantly, the product displayed superparamagnetic behaviour at room temperature with a more than 20% higher saturation magnetization.

## Findings

Superparamagnetic iron oxide nanoparticles (SPION) have a wide range of applications in biomedical research and development. The main drawbacks of SPION are a high surface energy, van der Waals forces of attraction and dipole to dipole interactions that cause them to agglomerate in ionic solution [1]. In addition, SPION exhibit a lack of affinity for biomolecules. One of the methods used to minimize these effects is through surface modification or functionalization of the SPION.

Organic compounds, such as (3-aminopropyl)triethoxysilane (APTES), are among the common materials with which SPION can be functionalized. APTES is a coupling agent and in addition can prevent the agglomeration of the SPION through steric repulsion. The terminal amine group of the aminosilane molecule is suitable for bioconjugation [2–4]. Moreover, the amine moiety is used as linker in the synthesis of composite or hybrid nanoparticles consisting of SPION and other inorganic materials such as gold nanoparticles [5–6]. More importantly, for targeting and delivery purposes the functional amine moiety can further be modified with other functional groups, such as peptides, antibodies, oligonucleotides or polymers [7–13].

As illustrated in Table 1, through a conventional method APTES can be grafted onto SPION by stirring, heating or combination of both heating and stirring. The combined heating and stirring process demonstrated to be the fastest method so far. However, in this letter we present a new, facile and rapid sonochemical method of synthesizing highly magnetic APTES-functionalized SPION. The sonochemical system consists of both mechanical vibration (stirring) and localized hot spot plus other unique conditions generated via acoustic cavitation process. These exceptional conditions are employed to sonochemically graft APTES on SPION.

**Table 1:** Comparing the conventional methods of grafting APTES on SPION.

author	methodology	reaction time (h)	saturation magnetization (emu/g)	reference

Ma et al.	rapid stirring	7	—	[14]
Yamaura et al.	heating with water bath	3	63.54	[15]
Shen et al.	heat and stirred at 40 °C	1	62	[16]

SPION were prepared through a co-precipitation method as reported in our previous work [17]. Because a huge amount of thermal energy is generated during the sonochemical process, the SPION–APTES compound was synthesized in an ice bath for heat dissipation. The colloidal suspension of SPION was initially dispersed for 2 min by using a Vibra-Cell ultrasonic horn. Subsequently, APTES was then added and the mixture was further sonicated for 20 min. The resulting product was left overnight and then separated with magnets (1.5 T, for details see Supporting Information File 1).

The ultrasonic irradiation of the mixture causes the formation, growth and collapse of bubbles (acoustic cavitation process) within the liquid content. These bubbles behave as individual microreactors as they are often accompanied by a violent collapse from which large amounts of energy are released [18]. The collapsed bubbles generate a high temperature, pressure, and cooling rate of up to 5000 K, 2000 atm and 10^10^ K/s, respectively [19]. In addition to the localized hot spot generated from the cavitation process, shock waves or microjets with huge pressure and high speed are produced, which are transferred into the liquid solution, thereby creating a mechanical stirring effect [20].

**Scheme 1:**
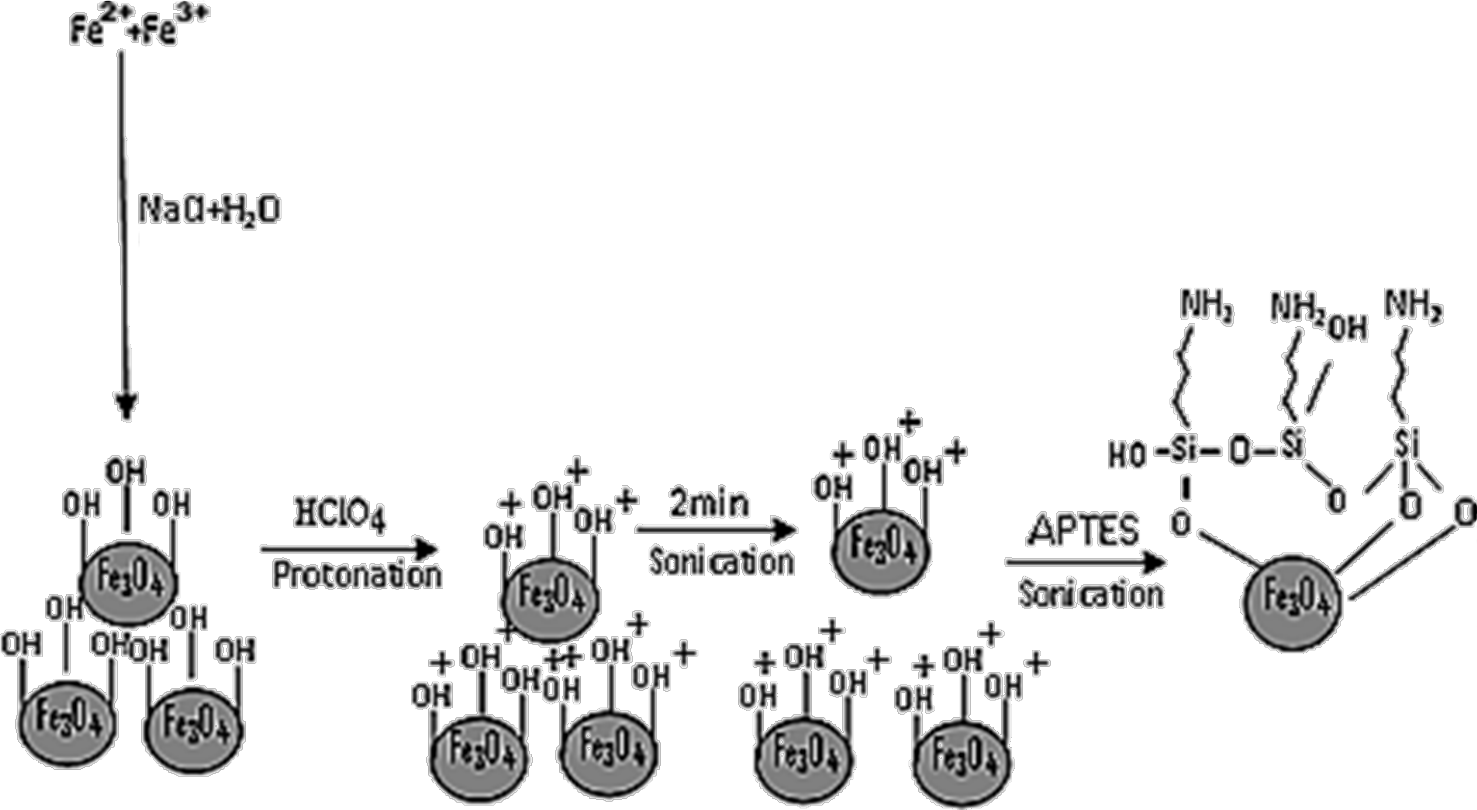
Schematic representation of the sonochemical synthesis of APTES-functionalized SPION.

As demonstrated in Scheme 1, these unique conditions generated from the sonochemical environment are harnessed to produce a facile and rapid silanization reaction between the SPION and APTES. Therefore, the energy provided by the initial 2 min sonication period was employed to disperse the SPION. Further 20 minutes sonication of the dispersed SPION in the presence of APTES generated an ultrasound beam with a power of approx. 35 W, which provided the sonochemical conditions that facilitated the rapid interaction between the precursors. However, this is an ongoing research the scope of this letter does not cover optimizing the various synthesis parameters. Importantly, the available data is not enough to determine the mechanism of this sonochemically assisted silanization process. More fundamental research needs to be carried out.

**Figure 1:**
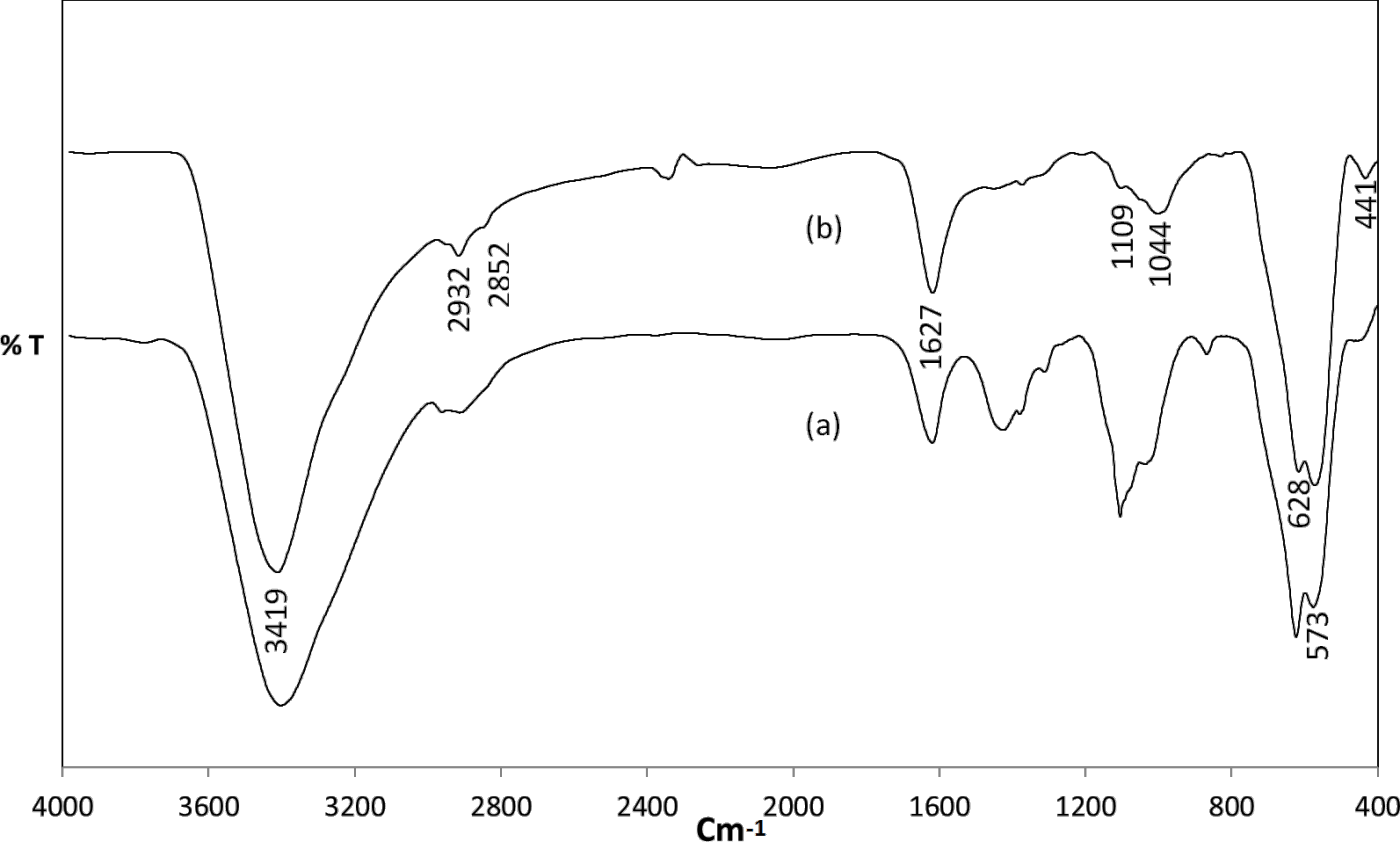
FTIR spectrum of (a) naked SPION (b) APTES-functionalized SPION. The binding of APTES onto the SPION is revealed by the 1109 and 1044 cm^−1^ peaks assigned to the Si–O–Si bond.

In the silanization reaction, the ethoxy groups of the APTES molecule react with the terminal hydroxy groups (OH) on the SPION binding site to form silanol (Si–O–H) groups. The Si–O–H further condenses with other silanol groups to form siloxane (Si–O–Si) bonds. The successful grafting of the APTES molecules on the SPION is verified through FTIR analysis (Figure 1). In both spectra, the peaks of the magnetite (Fe–O–Fe) band split into two. The energy absorbed at 628 and 573 cm^−1^ corresponds to the first band. The second band is observed at 441 cm^−1^ which is assigned to the Fe–O bond of bulk magnetite at 375 cm^−1^.

The formation of SPION–APTES is observed in Figure 1b by the 1109 and 1044 cm^−1^ peaks, which can be assigned to the Si–O–Si bond. The IR absorption lines due to the adsorption of water molecules to the surface of SPION via hydrogen bond can be observed by the appearance of the bands at 1627 and 3419 cm^−1^ in Figure 1a. Similar to the report of Ma et al., [14], the peaks of the N–H bending and stretching of terminal primary amine group cannot be seen in Figure 1b, as they overlap with the 1627 and 3419 cm^−1^ peaks, respectively. The absorption bands at 2852 and 2932 cm^−1^ in Figure 1b can be assigned to stretching vibration of the C–H bond of the propyl group present in APTES.

**Figure 2:**
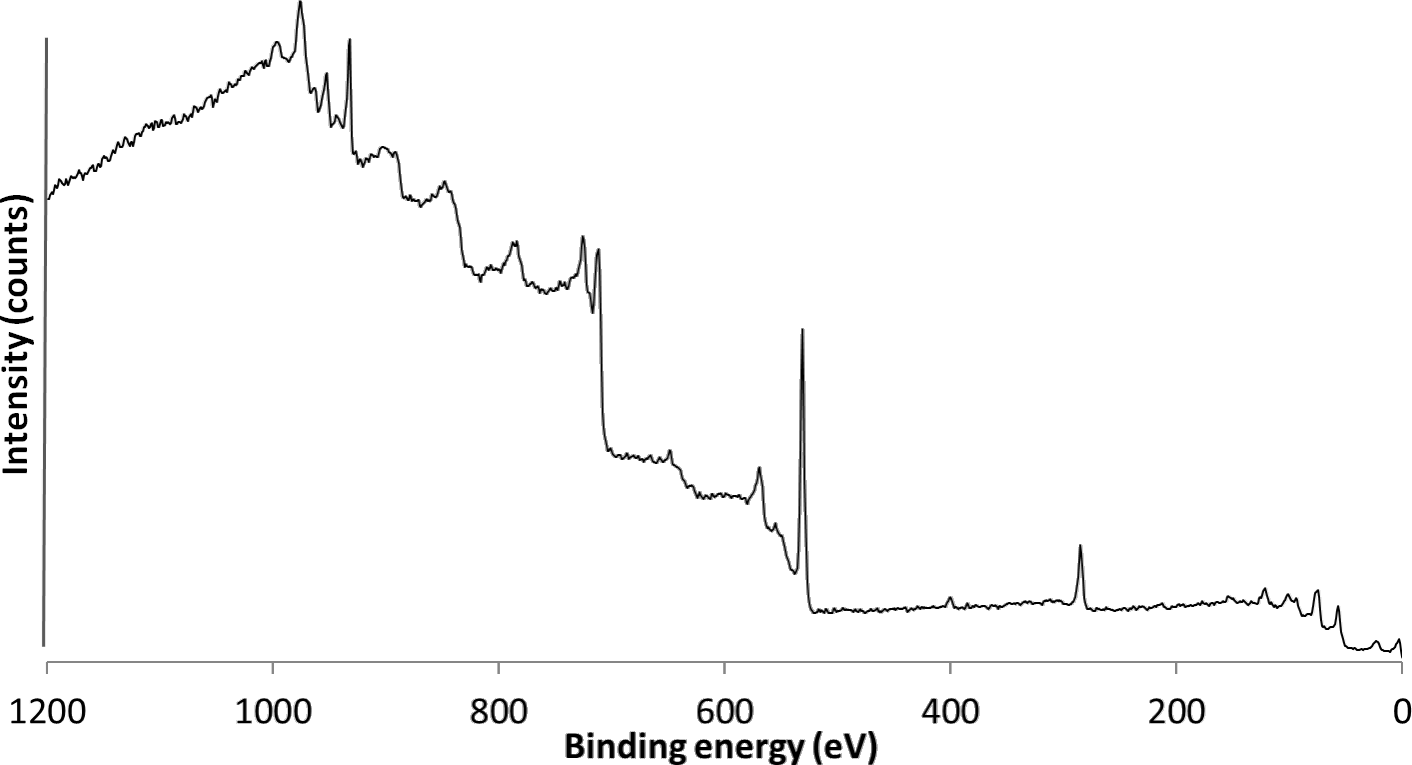
XPS spectrum showing APTES functionalized SPION, the bands at 103, 287 and 402 eV correspond to the presence of silicon, carbon and nitrogen on the surface of the SPION.

The XPS spectrum of the silanized SPION is shown in Figure 2. It further confirms the presence of Si, C and N on the surface of the SPION. The ionic states of these elements demonstrate the successful grafting of APTES on the SPION.

The various curve fitting graphs for the ionic state of each element are presented in Supporting Information File 1. The two peaks in the Si 2p curve fitting (Supporting Information File 1, Figure S1) corresponding to 102.5 eV and 103.8 eV can be assigned to the binding of Si to APTES and the formation of the Si–O–Si bond, respectively. The C 1s (Supporting Information File 1, Figure S2) shows three shifted bands at 285.3 eV, 287.8 eV and 289.97 eV which are assigned to aliphatic carbon in Si–CH_2_, -CH_2_–CH_2_, and -CH_2_–NH_2_, respectively. The O 1s peak (Supporting Information File 1, Figure S3) deconvolves into three bands at 531.32 eV, 531.59 eV and 534.14 eV that correspond to oxygen in Si–OH, Si–O and Fe–O. The N 1s (Supporting Information File 1, Figure S4) binding energy that reveals the characteristic of C–NH_2_ groups is absorbed by the weak 401.10 eV band. However, the weak peak is owing to low concentration of nitrogen in the APTES–SPION. This can be related to the amount of APTES used during the sonochemically assisted silanization reaction.

The presence of the SPION core can be observed by the band of Fe 2p3/2 and Fe 2p1/2 (Supporting Information File 1, Figure S5) that appears at 725.25 eV and 711.85 eV, respectively. The difference in their energy is 13.4 eV, which corresponds to 13.6 eV of Fe_2_O_3_ or Fe_3_O_4_. However, the XPS result alone cannot be used to determine the oxidation state of Fe in Fe_2_O_3_ or Fe_3_O_4_. This is due to similarity in the oxidation state of both iron oxide compounds. The chemical shifts observed in all the bands can be ascribed to the binding of the APTES on the SPION.

**Figure 3:**
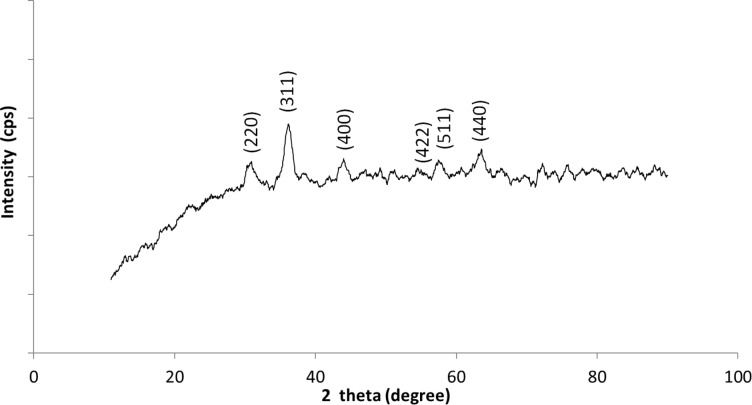
XRD pattern of APTES-functionalized SPION with the peaks correspond to either magnetite or maghemite compared with JCPDS 5-0664.

The XRD pattern of the silanized SPION is shown in Figure 3. It corresponds to the JCPDS standard of either magnetite or maghemite (cubic phase) XRD spectrum. However, the assignment to one of these phases on the basis of the XRD is difficult owing to their related structures. The absence of peaks at 110 and 104 corresponding to goethite and hematite in the spectrum indicate that the co-precipitation and the various sonochemical parameters considered in this work are optimized to synthesis silanized SPION.

**Figure 4:**
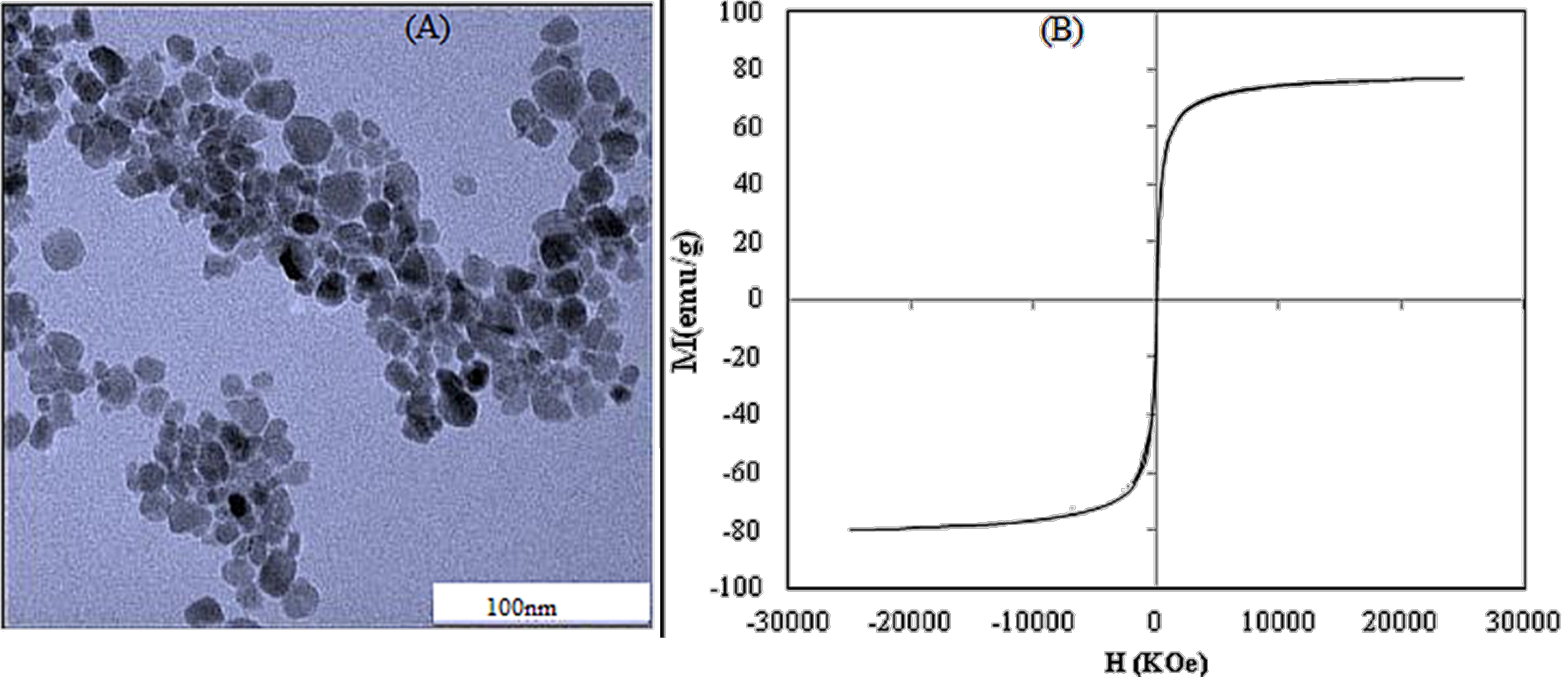
(a) TEM micrograph of the APTES–SPION (b) Magnetization curves of the APTES-functionalised SPION at 300 K.

The non-agglomeration of the SPION as shown in the micrograph (Figure 4a), can be attributed to the steric repulsion provided by APTES on the surface of the SPION. Like the magnetization curve presented in the work of Yamaura et al., our result (Figure 4b) shows a typical superparamagnetic behaviour. It showed no hysteresis and was completely reversible at 300 K. Neither coercivity nor remanence was observed. Our silanized SPION have a saturation magnetization value of 77.7 emu/g, which is higher compared to 62 emu/g and 63.54 emu/g reported in the work of Yamaura et al. and Shen et al., respectively [15–16].

## Conclusion

We presented a simple means of functionalizing SPION. In this work, the unique sonochemical conditions and energy generated by an acoustic cavitation process were employed to graft APTES on the surface of SPION. This coating approach has provided a rapid and an effective method of synthesizing highly magnetic silanized SPION.

## Supporting Information

Supporting Information contains the experimental section. In addition, information on the XPS and curve fittings for each element (Si, C, O, N and Fe) on the surface of the SPION are shown.

File 1 Experimental and XPS data.
