# Supplementary material for: A sonochemical approach to the direct surface functionalization of superparamagnetic iron oxide nanoparticles with (3-aminopropyl)triethoxysilane
Source: Beilstein J Nanotechnol. 2014 Sep 8;5:1472–6. doi: 10.3762/bjnano.5.160 (PMC4168857; doi:10.3762/bjnano.5.160)
Supplement: File 1 — Experimental and XPS data. [file Beilstein_J_Nanotechnol-05-1472-s001.pdf]

Supporting Information

for

A sonochemical approach to the direct surface functionalization of superparamagnetic iron oxide nanoparticles with (3-aminopropyl)triethoxysilane

Bashiru Kayode Sodipo* and Azlan Abdul Aziz

Address: Nano-Biotechnology Research and Innovation (NanoBRI), Institute for Research in Molecular Medicine (INFORMM), Universiti Sains Malaysia, 11800, Pulau Pinang, Malaysia and Nano-Optoelectronic Research and Technology Lab (NOR Lab), School of Physics, Universiti Sains Malaysia, 11800 Pulau Pinang, Malaysia

Email: Bashiru Kayode Sodipo* - bashirsodipo@gmail.com; Azlan Abdul Aziz - lan@usm.my

* Corresponding author

Experimental and XPS data

Materials and methods

Ferric chloride hexahydrate ($FeCl_3.6H_2O$ >99%), ferrous chloride tetrahydrate ($FeCl_2.4H_2O$ >99%), sodium chloride salts, sodium hydroxide, 3-aminopropyltriethoxysilane (APTES 99%) were bought from Sigma-Aldrich and used directly without any further purification. 20 kHz Vibra-Cell ultrasonic with 13 mm diameter horn was used for the sonochemical reactions.

Synthesis of SPION

SPION was prepared according to co-precipitation method as reported in our previous work [1]. Briefly, in an inert atmosphere of nitrogen gas, room temperature and apparent pH 10, 1 M of $FeCl_2$ and 2 M of $FeCl_3$ solutions were co-precipitated with 1 M sodium chloride solution. The system was left to agitate for 2 hours at stirring of 500 rpm. The magnetic nanoparticles were collected in a beaker and the supernatant was removed. The particles were then washed thoroughly with distilled water to remove unreacted chemicals. To temporarily prevent agglomeration of the magnetic nanoparticles, the SPION was peptized with 3.5 M perchloric acid ($HClO_4$) for 16 hours. Supernatant of the sample was removed. The peptized SPION was finally dispersed in distilled water.

Sonochemical synthesis of APTES functionalized SPION

The amplitude of the sonicator was adjusted to 47% and then in an ice bath environment, a 20 mL of the colloidal suspension of SPION was initially ultrasonically irradiated (dispersed) for 2 minutes. Subsequently, 5 mL APTES (99%) solution was added and further sonicated for 20 minutes. The resulting product was left overnight and subsequently separated with 1.5 T magnet. The prepared composite nanoparticles were washed thoroughly with distilled water and dried in an oven to produce a fine powdery form.

Characterization

Fourier transforms infrared (FT-IR) spectroscopy Perkin Elmer System 2000, was used to examine the binding of APTES on the SPION. The surface elements were determined using high resolution multi technique X-Ray photoelectron Spectrometer (Axis Ultra DLD XPS, Kratos). The X-ray diffraction (XRD) of the product was characterized with D/max-IIIC X-ray diffractometer (Shimadzu, Japan). The peaks were compared to the JCPDS 5-0664 of International Centre for Diffraction Data to determine the crystalline structures. The morphology and size of particles were determined through transmission electron microscopy (TEM) Zeiss Libra 120. The magnetization measurement was obtained at room temperature in magnetic field up to 10 kOe using a vibrating sample magnetometer.

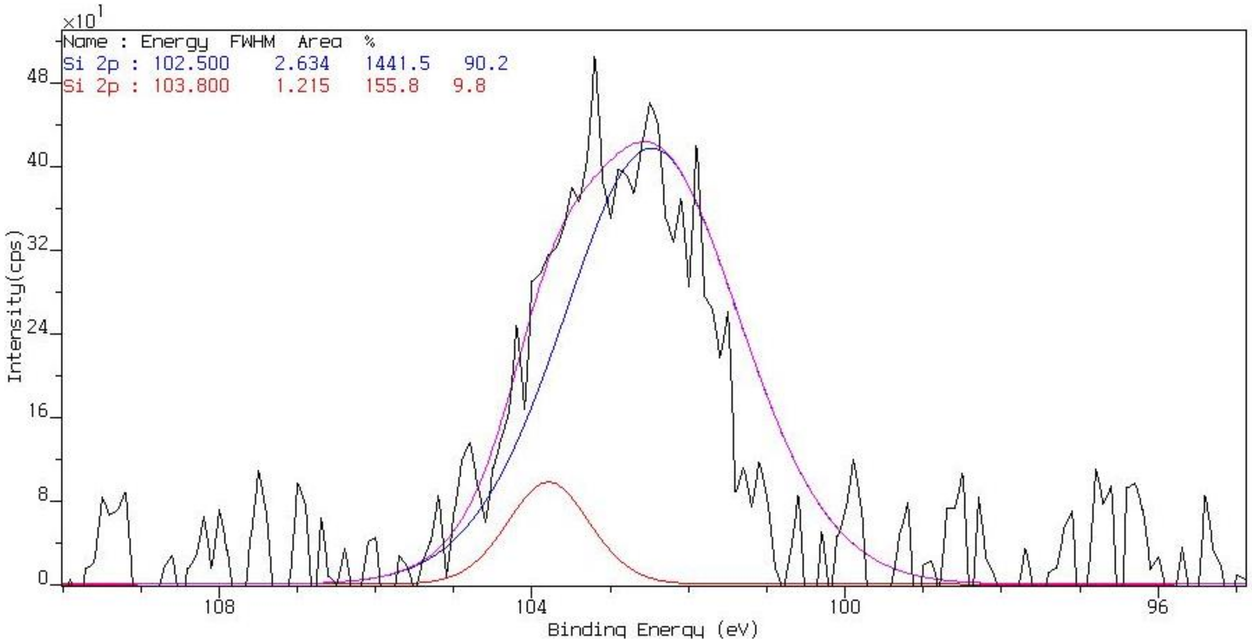

Figure S1: Showing the binding energy and ionic state of Si present in the APTES functionalized SPION.

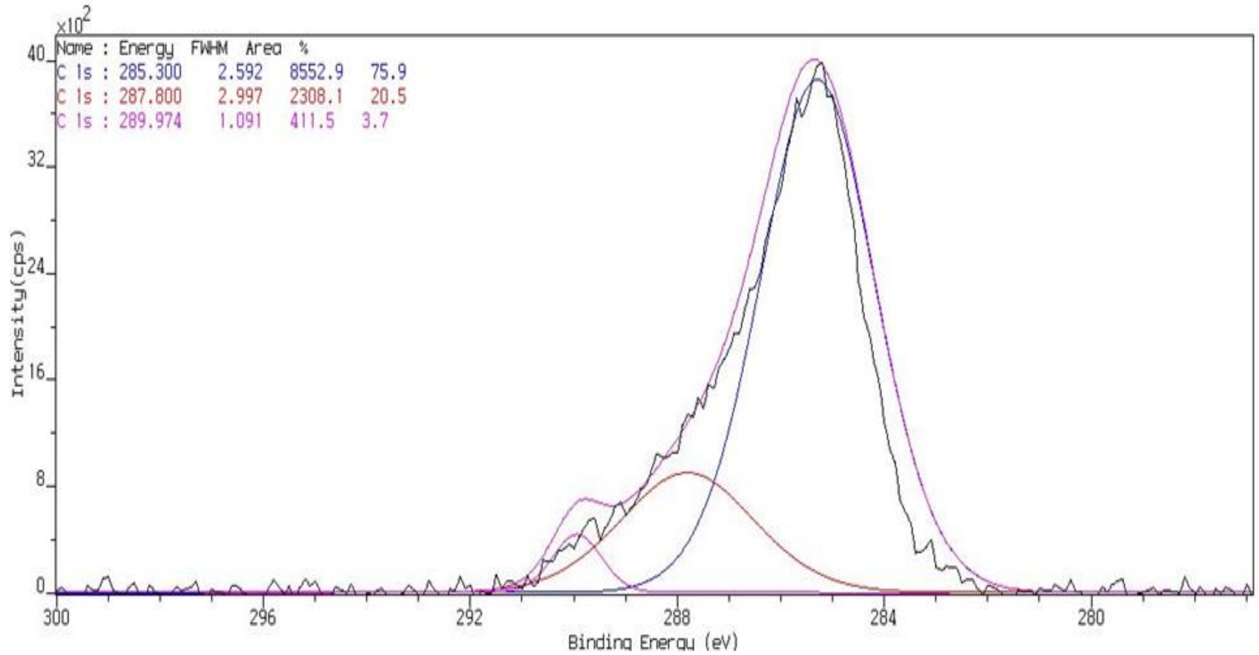

Figure S2: Showing the binding energy and ionic state of C present in the APTES functionalized SPION.

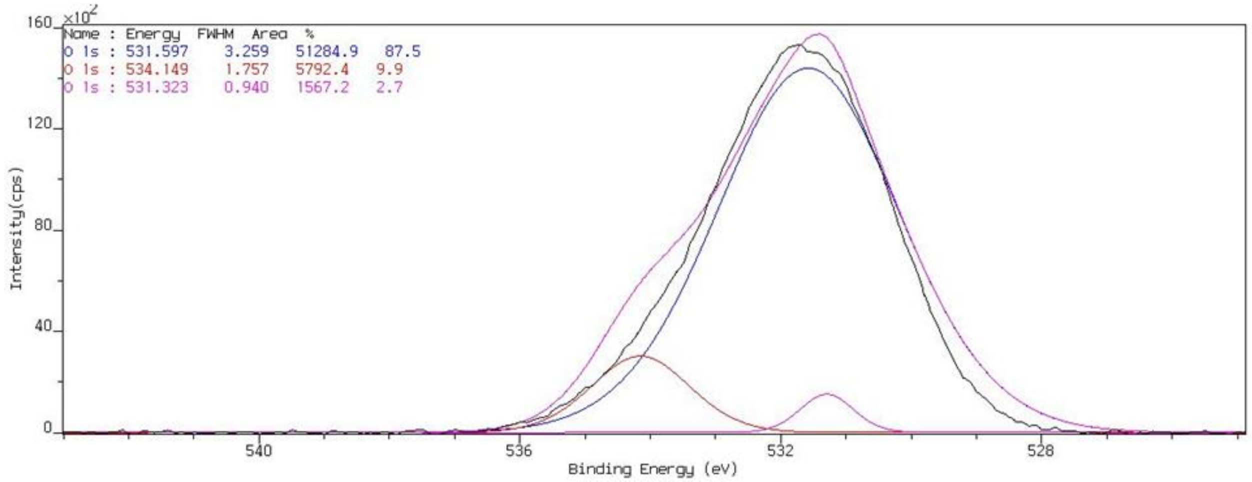

Figure S3: Showing the binding energy and ionic state of O present in the APTES functionalized SPION.

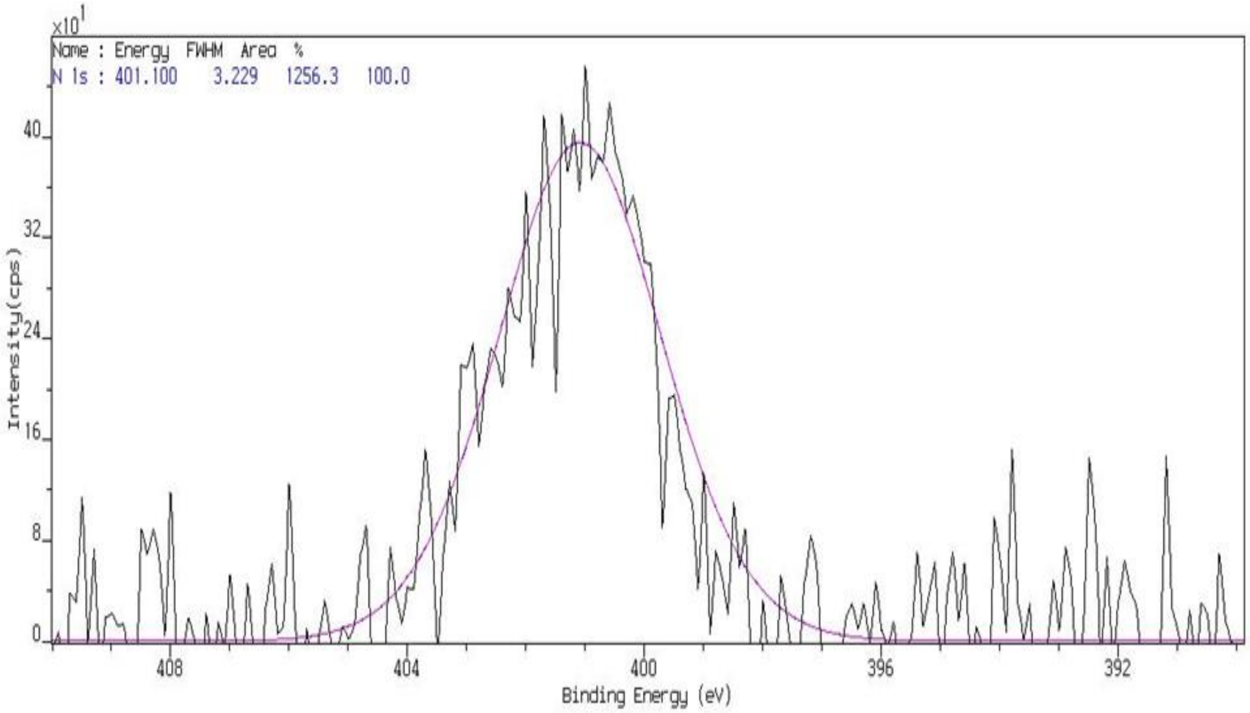

Figure S4: Showing the binding energy and ionic state of N present in the APTES functionalized SPION.

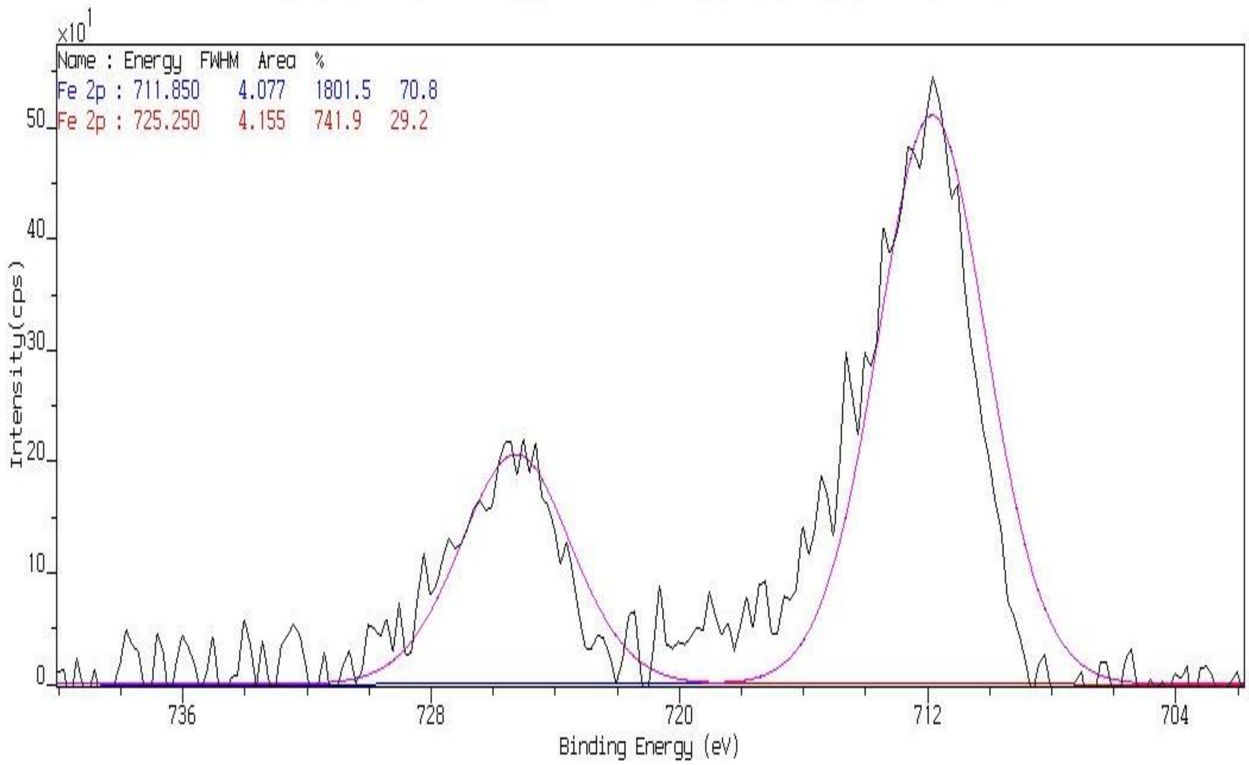

Figure S5: Showing the binding energy and ionic state of Fe present in the APTES functionalized SPION.

References

1. Sodipo, B. K.; Aziz, A. A. Sonochemical synthesis of silica coated superparamagnetic iron oxide nanoparticles. In *Materials Science Forum, Vol. 756;* Trans Tech Publishing, 2013; pp 74–79.
